# Maternal Transmission Ratio Distortion in Two Iberian Pig Varieties

**DOI:** 10.3390/genes11091050

**Published:** 2020-09-05

**Authors:** Marta Vázquez-Gómez, Melani Martín de Hijas-Villalba, Luis Varona, Noelia Ibañez-Escriche, Juan Pablo Rosas, Sara Negro, José Luis Noguera, Joaquim Casellas

**Affiliations:** 1 Departament de Ciència Animal i dels Aliments, Universitat Autònoma de Barcelona, 08193 Bellaterra, Spain; Melani.MartinDeHijas@uab.cat (M.M.d.H.-V.); joaquim.casellas@uab.cat (J.C.); 2 Departamento de Anatomía Embriología y Genética Animal, Universidad de Zaragoza, 50013 Zaragoza, Spain; lvarona@unizar.es; 3 Departament de Ciència Animal, Universitat Politècnica de València, 46071 València, Spain; noeibes@dca.upv.es; 4 Programa de Mejora Genética ‘Castúa’, Inga Food SA, 06200 Almendralejo, Spain; juan.rosas@nutreco.com (J.P.R.); snegram@gmail.com (S.N.); 5 Genètica i Millora Animal, Institut de Recerca i Tecnologia Agroalimentàries, 25198 Lleida, Spain; JoseLuis.Noguera@irta.cat

**Keywords:** genome scan, maximum likelihood, segregation distortion, *Sus scrofa*, ungenotyped parents

## Abstract

Transmission ratio distortion (TRD) is defined as the allele transmission deviation from the heterozygous parent to the offspring from the expected Mendelian genotypic frequencies. Although TRD can be a confounding factor in genetic mapping studies, this phenomenon remains mostly unknown in pigs, particularly in traditional breeds (i.e., the Iberian pig). We aimed to describe the maternal TRD prevalence and its genomic distribution in two Iberian varieties. Genotypes from a total of 247 families (dam and offspring) of Entrepelado (*n* = 129) and Retinto (*n* = 118) Iberian varieties were analyzed. The offspring were sired by both ungenotyped purebred Retinto and Entrepelado Iberian boars, regardless of the dam variety used. After quality control, 16,246 single-nucleotide polymorphisms (SNPs) in the Entrepelado variety and 9744 SNPs in the Retinto variety were analyzed. Maternal TRD was evaluated by a likelihood ratio test under SNP-by-SNP, adapting a previous model solved by Bayesian inference. Results provided 68 maternal TRD loci (TRDLs) in the Entrepelado variety and 24 in the Retinto variety (*q* < 0.05), with mostly negative TRD values, increasing the transmission of the minor allele. In addition, both varieties shared ten common TRDLs. No strong evidence of biological effects was found in genes with TRDLs. However, some biological processes could be affected by TRDLs, such as embryogenesis at different levels and lipid metabolism. These findings could provide useful insight into the genetic mechanisms to improve the swine industry, particularly in traditional breeds.

## 1. Introduction

Transmission ratio distortion (TRD) occurs when two alleles at a heterozygous locus are not transmitted equally to the offspring, disrupting Mendelian segregation (0.5 probabilities of transmission; [1,2,3]). The TRD phenomenon has been reported in humans [4,5,6], other mammals [3,7], insects [8,9], and even in plants [10,11]. Different biological mechanisms can cause TRD before or after fertilization [12,13,14,15,16]. In mammals, there are different mechanisms in sires and dams prior to fertilization during the meiotic or gametic stages. For example, the meiotic drive is restricted to female gametes (asymmetry meiosis), whereas male gametes (symmetric meiosis) have gamete competition [13,17]. In this way, some causes of TRD could not be studied depending on the parents’ data available. Afterward, there are potential mechanisms of distortion during the zygotic stage, such as asymmetric hybrid incompatibility. Thus, it is currently challenging to isolate the stage at which TRD occurs and pinpoint a biological cause. However, the study by sex is often the first and most accessible step to find the underlying mechanism.

In pigs, there is a lack of information about the TRD phenomenon, although it could induce bias in genetic mapping studies [16,17,18,19]. The few references about pigs are mainly related to a specific locus in genes or other kinds of segregation distortions [20,21,22]. So far, the greatest TRD study in pigs is the mapping of TRD loci (TDRLs) in boars and part of their offspring using a Bayesian approach to detect paternal TRDLs in the pig genome, but no maternal TRDLs [23]. There are no studies in pigs to assess the segregation deviation by standard χ2 [4,24], t-tests [25], or likelihood ratio tests [18,26].

The lack of information about the TRD phenomenon is more considerable in traditional swine breeds, such as the Iberian breed, because of their lower commercial interest and the scarcity of their organized breeding programs compared to modern swine breeds [27,28,29]. However, the interest in these traditional breeds is increasing in recent decades due to their high-quality pork products and socio-economic effects, and the Iberian breed is one of the most important worldwide from an economic point of view [30,31,32]. Traditional swine breeds also have high genetic diversity [33,34,35]. The Iberian breed consists of small populations with remarkable phenotypic and genomic differences categorized in varieties and strains that currently are crossed between them due to commercial goals, such as higher prolificacy and neonatal survival, and better productive indexes [36,37,38]. Thus, mapped TRDLs in these kinds of breeds can accelerate both the detection of TRD and the characterization of their underlying genetic mechanisms. Moreover, these data could provide insight into genetic and evolutionary mechanisms of individual fitness variation and population divergence for improving the current Iberian swine production [39].

In this study, we aimed to describe the maternal TRD phenomenon prevalence and its genomic distribution in two varieties of the Iberian pig breed. Moreover, we have developed an adaptation of a Bayesian model [23] using a different statistical approach to detect TRDLs.

## 2. Materials and Methods

### 2.1. Animals, Data, and Ethics Statements

The management of animals was assessed and approved by the IRTA Committee of Ethics in Animal Research (Institute of Agrifood Research and Technology, Caldes de Montbui, Spain; IRTA-22032012). It was performed according to the Spanish Policy for Animal Protection (RD 53/2013), which meets the European Union Directive 2010/63/UE on the protection of research animals.

A total of 109 purebred Entrepelado (*n* = 52) and Retinto dams (*n* = 67) and their 247 daughters were sampled from two selection farms located in Extremadura (southwestern Spain). While all dams were purebred, daughters were either purebred or F_1_ crosses with the alternative Iberian variety (i.e., daughters from Entrepelado dams were purebred Entrepelado and Entrepelado-by-Retinto F1 crosses). Both Retinto (R) and Entrepelado (E) varieties of the Iberian pig breed are officially recognized by Spain’s Iberian herd book (AECERIBER, Zafra, Spain). Animals belonged to commercial breeding stocks founded in 2009 by Inga Food SA (Almendralejo, Spain). Biological samples were collected from blood or tail tissue and kept frozen until laboratory processing. Procedures for DNA extraction relied on standard phenol-chloroform (with a preliminary 24-h digestion by proteinase K) for tail samples, whereas the Invisorb^®^ Blood Mini HTS 96 kit/C (INVITEK Molecular GmbH, Berlin, Germany) was used for blood samples. Quality control for DNA samples was performed by a NanoDrop™ 2000 (Thermo Fisher Scientific, Waltham, MS, USA) and Qubit Fluorometer (Thermo Fisher Scientific) with a minimum DNA concentration of 40 ng/μL. All animals were genotyped with the Geneseek Genomic Profiler Porcine HD (Illumina, Inc., San Diego, CA, USA; 70,231 SNPs) at the Centre for Research in Agricultural Genomics (CRAG, Bellaterra, Barcelona, Spain).

A total of 247 families (each dam and each genotyped daughter, in our case) were used for TRD analyses. Families were classified, first, by the maternal Iberian pig variety and, second, by the paternal Iberian pig variety. Among the Entrepelado dam families, 97 were from crosses with Entrepelado sires (EE) and 32 from crosses with Retinto sires (RE). On the other hand, among the Retinto dam families, 57 were from crosses with Entrepelado (ER) sires and 61 from crosses with Retinto sires (RR). Entrepelado and Retinto dam families were independently analyzed for the TRD analyses, as described below. Final analyzed genetic markers were obtained after filtering [23], among a total of 63,060 single-nucleotide polymorphisms (SNPs) mapped in the *Sus Scrofa* 11.1 genome map. Before TRD analyses, all SNPs with a minimum allele frequency (MAF) and a call rate of 0% (14,527 and 3046 SNPs, respectively) were removed to improve analysis time and resources. After TRD estimations, SNPs were filtered based on the family consistency rate >75% (families with complete information; Entrepelado, 31,286; Retinto, 29,696 SNPs). Second, they were filtered by a minimum allele frequency of >5% (Entrepelado, 18,681; Retinto, 11,857 SNPs) and, finally, by within-variety dam heterozygosity of >20%. This last parameter is particularly important in TRD analyses because families with a heterozygous dam are useful to calculate the distortion estimate (described below in the model description). Only 16,246 (25.8%) were used in final analyses for the Entrepelado variety and 9744 (15.5%) for the Retinto variety.

### 2.2. Transmission Ratio Distortion Analysis

The model used in the current study to identify the maternal TRD phenomena in both maternal Iberian breed varieties, Entrepelado and Retinto, was a frequentist modification of an allelic TRD model [23] allowing the use of ungenotyped ancestors within the TRD framework, which was based on another method [24].

In brief, the original model assumes biallelic genetic markers (alleles A1 and A2) and presents an inheritance model, where the probability of each genotype in the offspring is defined by the inheritance probability of a given allele from the sire and the dam. Assuming a heterozygous dam and an ungenotyped sire, probabilities of inheritance for all three plausible genotypes in the offspring were assumed:

*p*_offspring_ (A1A1) = *p*_dam_ (A1) *p*_sire_ (A1)

*p*_offspring_ (A1A2) = *p*_dam_ (A1) *p*_sire_ (A2) + *p*_dam_ (A2) *p*_sire_ (A1)

*p*_offspring_ (A2A2) = *p*_dam_ (A2) *p*_sire_ (A2)

where allele probabilities could be easily defined for ungenotyped sires,

*p*_sire_ (A1) = π = 1 − *p*_sire_ (A2)

and genotyped and heterozygous dams,

*p*_dam_ (A1) = 0.5 + α

*p*_dam_ (A2) = 0.5 – α

Note that π captured A1 frequency in the sire population and ranged between 0 and 1. On the other hand, α was the estimate of the dam-specific TRD parameter and took values between −0.5 and 0.5. In this specific case, the maternal TRD estimates are assumed to be free from sire-specific TRD influences (they would be masked within the π parameter of sire; [23]). Allele probabilities from A1A1 homozygous dams were straightforwardly defined as

*p*_dam_ (A1) = 1

*p*_dam_ (A2) = 0

and the same rationale was applied to A2A2 dams.

In our study, we independently analyzed Entrepelado and Retinto dam families. Within each scenario, daughters were both purebred and F1 crosses. So, two independent π parameters were required to accommodate the genetic background of the sire (i.e., πE for Entrepelado sires and πR for Retinto sires). Consequently, previous expressions were modified as follows for offspring for Entrepelado sires,

*p*_sireE_ (A1) = πE = 1 − *p*_sireE_ (A2)

and Retinto sires,

*p*_sireR_ (A1) = πR = 1 − *p*_sireR_ (A2)

Unknown parameters (α, π_E_, π_R_) were estimated for each population by maximizing the likelihood function under a three-dimension gradient ascent approach. Statistical significance for α was evaluated through a standard likelihood ratio test [40] under an SNP-by-SNP approach, as other previous TRD models have used [18,26,41,42]. Ad hoc software was written in FORTRAN90 programming language. The A1 allele in our program was always the allele with the higher frequency, so all TRDL results have the same pattern. Subsequent analyses and figures were carried out using R software [43]. Maternal TRD results were corrected for multiple testing using the false discovery rate approach (FDR; α = 0.05; *q*-*values* [44]).

### 2.3. Analysis of Gene Enrichment

Common genetic markers with significant TRD values (*q* > 0.05) were used for the analysis of gene enrichment. Genes within a window of 500 Kb around each selected region (upstream and downstream) were identified using the Ensembl BioMart tool with the *Sus scrofa* 11.1 genome map [45]. These genes were directly used to identify enriched Gene Ontology (GO) terms for biological processes in *Sus scrofa* as the reference genome with the Panther Tool using the PANTHER GO-slim v15.0 dataset [46]. Results were corrected using Bonferroni correction for multiple testing. Moreover, genes with TRDLs were found in the human and animal datasets of Mendelian disorders [47,48].

### 2.4. Data Availability

Raw *p*-values, maternal TRD, and sire frequency estimates of both family varieties from SNPs analyzed are available (Table S1).

## 3. Results and Discussion

Ungenotyped ancestors can be a condition in research studies due to their characteristics of old valuable datasets, previous designs, or complications during the development of studies. These missing genotypes in the parental generation can become a major problem using previous methods that need known expected frequencies in the last generation or offspring genotypes anticipated by the experimental cross itself [24,25]. Our modified multinomial model based on an original method [23] allowed us to study maternal TRDLs in swine, with only offspring and dam data. Moreover, the change in the statistical approach of our model allows more straightforward interpretations of results and is more accessible to researchers or end-users not familiarized with Bayesian inference. This statistical method could also help to implement another statistical approach in the future, as evidential inference. Thanks to the flexibility of our model, we have not only analyzed TRDLs in families without sire data, but also we have analyzed, together, family datasets from different varieties of the same breed without giving expected frequencies.

### 3.1. Estimates of Maternal Transmission Ratio Distortion Loci

Using our model, we have analyzed a total of 21,803 SNPs (34.5%, after filtering described in Material and Methods) from both Entrepelado and Retinto dam families to find TRDLs among the Iberian pig genome. From this final SNP list, only 4187 SNPs (6.6%) analyzed were in datasets of both maternal varieties due to different levels in the dam heterozygosity of each Iberian variety by SNP. Nevertheless, the dam heterozygosity % (mean, min-max) was similar between the Entrepelado (45.6, 21.19–100%) and Retinto (43.45, 21.19–100%) dam family datasets used. Another cause of differences between both varieties could be that the Retinto variety is considered more ancient than the Entrepelado variety, even being a possible origin of the Entrepelado variety [49,50,51,52]. Moreover, the Retinto variety founders from both farms used in our study had a single origin, while the Entrepelado variety founders had four different origins (personal communication, E Mazallón). All these factors could affect heterozygosity, as we could see after filtering. The Retinto variety lost two-thirds of available SNPs, while the Entrepelado variety lost almost half. Proper filtering of bad markers (high counts of missing genotypes, non-informative markers), before the TRDL search, is important to avoid loci distorted for non-biological reasons [16]. Notably, this step was essential with our low sample size, after dividing the total dataset by the maternal Iberian variety, to decrease the risk of overestimating TRDL effects above a given statistical threshold.

**Figure 1:**
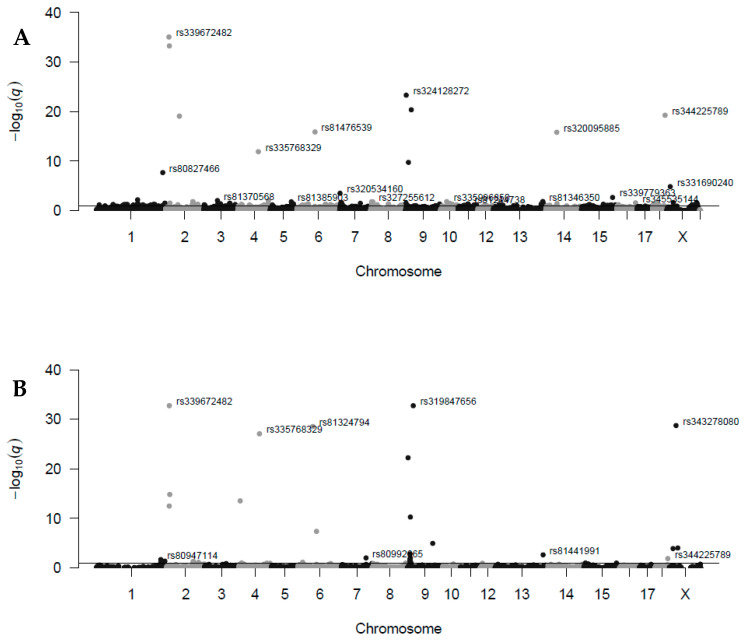
Distribution of maternal transmission ratio distortion loci (TRDLs) in families of Entrepelado (**A**) and Retinto (**B**) varieties across pig chromosomes. The top hit marker ID on each chromosome below the *q*-value threshold (0.05, corrected *p*-value; line) is annotated.

A higher amount of maternal TRDLs were found in the Entrepelado (68 TRDLs, *q* > 0.05; Table S2) than the Retinto variety (24 TRDLs, *q* > 0.05; Table S3) among the whole genome (Figure 1), with distances between TRDLs in the same chromosome between 3.8 Kb and 99.1 Mb in both varieties. The Entrepelado variety also showed a more extensive distribution (Figure 1A) than the Retinto variety (Figure 1B), with TRDLs in all chromosomes except 12 and 17. In contrast, the Retinto variety only had TRDLs in chromosomes 1, 2, 4, 6, 7, 9, 13, 18, and X. This could be related to the greater amount of TRDLs found in the Entrepelado variety. In a unique previous study of TRDLs among the whole pig genome, chromosome 12 was the only one without TRDLs [23]. Nevertheless, this chromosome is the smallest in the pig genome and has the lowest amount of SNPs, being a critical point to find TRDLs. On the other hand, the minimum dam heterozygosity percentage of detected maternal TRDLs was 30.2 in the Entrepelado variety (mean: 59.5, max: 100%) and 44.5 in the Retinto variety (mean: 59.5, max: 100%). These values are greater than our cut-off limit of the dam heterozygosity percentage to filter SNPs (20%). Thus, our cut-off point did not alter the search for possible TRDLs. This overall description of both Entrepelado and Retinto maternal TRDL sets must be carefully interpreted because we could think that the Entrepelado variety has more TRD phenomena than the Retinto variety. However, we have to take into account that the Entrepelado variety dataset analyzed was larger than the Retinto variety dataset (discussed above). Even almost 60% of significant Entrepelado variety TRDLs were not in the Retinto variety dataset. In a previous study carried out with a final dataset of nearly 30,000 SNPs from five half-sib families and 352 offspring, there were 84 significant TRDLs with between 20 and 80% heterozygous sires [23]. So, it seems clear that the larger the marker dataset, parent heterozygosity, and sample size, the greater the potential to find TRDLs, as expected [16]. 

**Figure 2:**
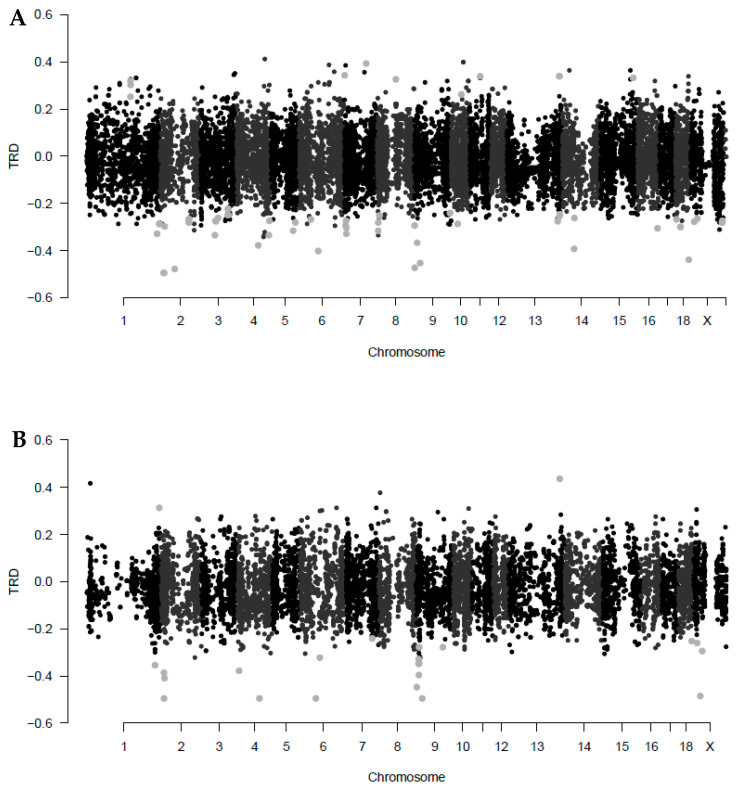
Distribution of maternal transmission ratio distortion (TRD) values in families of Entrepelado (**A**) and Retinto (**B**) varieties across pig chromosomes. Light grey points are significant TRD (*q* < 0.05).

Regarding TRD estimates (Figure 2), most of them were concentrated around 0, between −0.15 and 0.15. The Entrepelado variety showed wider ranges of TRD (0.252–0.393 and −0.223–−0.495; Table S2) than the Retinto variety (0.313–0.436 and −0.241–−0.495; Table S3), particularly in the positive values. The amount of significant negative TRD values was greater than the significant positive TRD values in both Entrepelado (84%; Figure 2A) and Retinto (92%; Figure 2B) varieties. Therefore, there would be more Mendelian TRDs promoting the minor allele than the greatest frequency allele in our sample, according to our method of analysis. However, there were more positive TRD values than negative in the study of Casellas et al. (2014) and with lower minimum absolute TRD values than in our results. No significant common TRDLs were found between this previous study and our results. Neither TRDLs were found in the *IGF2* locus (also described in humans) and the histocompatibility antigen loci [20,21,53]. Even though these results were in other pig breeds, it is important to consider that our study has analyzed datasets from dams but not sires. Although isolating the stage or sex at which TRD occurs is rarely enough to find a biological cause, it is often the first step to know the underlying mechanisms. The TRD phenomenon can result from a variety of selective processes during meiosis, gametogenesis, fertilization, and early zygote development [13,14,16,17,54]. In our study, it is possible to discard sperm competition from males as a cause, but not a distortion during chromosomal competition during meiosis or ovule competition from females: neither fertilization nor zygotic mechanisms. Therefore, further research using analyses with both parents (trios) and separately could be interesting to distinguish the specific maternal and paternal (meiotic/gametic mechanisms) influences and zygotic mechanisms on TRD.

Among TRDLs of both maternal Iberian varieties, only 10 of them were common (Table 1). These TRDLs were in six different chromosomes and separated at least by 1.8 Mb. Their range of dam heterozygosity (mean: 90.2, 58.5–100%) was lower than in separated TRDLs by the maternal Iberian variety, and TRD values were always negative (−0.252–−0.495) and roughly similar between maternal varieties. Although these varieties belong to the same breed, phenotypic and genomic differences between Iberian populations are remarkable due to the genetic drift and a scarce genetic flow [34]. In this situation, inbreeding depression may cause single-locus TRD at the zygotic level, affecting the homozygosity of lethal or highly deleterious recessive alleles that reduce biological fitness. This TRDL would make the purge of this kind of allele difficult. Indeed, inbreeding depression has already been reported in most of the Iberian pig varieties [39,52,55]. Among the common genes with at least one TRDL, none were related to lethal recessive alleles. Indeed, most of the TRDLs detected overall in the current study are intron variants. However, we have found a missense variant in an *olfactory receptor 5B2-like* gene and a TRDL in the *ADCY10* gene, associated with hypercalciuria in humans [47,56].

Intra-population TRD may be useful to measure inbreeding depression and study diversity. In our result, there are more negative TRD values, meaning there is an increase in the transmission of the minor allele. Evidence in humans showed that minor alleles are more likely to be risk alleles on complex diseases, so this could affect their physiology [57,58]. However, there are other possible links to allele frequencies. One could be their association with possible adaptations of the Iberian breed, traditionally reared in extensive conditions, to its new environment in intensive rearing conditions, as an environmental selection. Another could be an increase in genetic drift because of the increase in both varieties’ populations in recent decades [51,59]. On the other hand, inter-population TRD, such as our study, could lead to the wrong inference of meiotic drive or fertilization, but by increasing the tracking power of grandparental alleles using next-generation genotyping approaches, this will likely decrease [16,60]. Moreover, the TRD phenomenon can influence genetic mapping studies by reducing the sample size of informative genotypes and bias quantitative trait locus (QTL) estimate [18,19]. Therefore, increasing knowledge about this phenomenon may help to improve breeding programs and conservation strategies in the future, particularly for traits linked to TRD phenomena. However, further research is still necessary to understand the TRD phenomenon better. In this way, our model could be used to take advantage of all the data generated for genomic selection to analyze the TRD phenomenon as a novel trait with likely important implications for the livestock industry, especially from the reproductive point of view.

### 3.2. Possible Biological Implications of Transmission Ratio Distortion Loci

Both Entrepelado and Retinto varieties showed some TRDLs in genes (Table 2) with inherited disorders described in humans and/or animals, although there are no data of TRD for them [47,48]. In the Entrepelado variety, there are some genes with at least one TRDL related to different disorders in humans, such as *NUP214* (leukemia, 2 TRDLs), *GCNT2* (cataracts, 2 TRDLs), and *DPP6* (Ventricular fibrillation). The *ATP8B1* gene, associated with cholestasis and hepatic injury in mice, also had two TRDLs. Finally, *EVC2,* with one TRDL, is related to Ellis van Creveld syndrome, a chondrodysplasia detected in bovines, humans, dogs, and pigs. On the other variety (Retinto), *ZFAT* (autoimmune thyroid disease) and *CD36* (vascular diseases and the thrombospondin receptor in goats) genes had a TRDL. The *ZFAT* gene can also affect early embryonic lethality and placenta development in rodents. We have found several candidate genes susceptible to inherited disorders due to variant changes, but we can predict no clear implications of the TRDLs found. Besides, some TRDLs are close, so further analyses with larger sample sizes and useful marker datasets could improve this kind of approach and add the evaluation of linkage loci, such as using a haplotype model [16,61,62].

Another described cause of TRD could be the unconscious selection, the environmental selection, especially in inter-population or interspecific crosses. This idea could add a biological reason for the differences found between TRDLs of this study and previous studies in different pig breeds. The analysis of genes from common regions containing TRDLs in both Iberian varieties showed different enriched categories (Table 3), mainly related to developmental processes in embryos, such as morphogenesis, axogenesis, and neural development. Most of these GO biological processes or associations with the same parental lineages of embryogenesis were also found separately enriched in Entrepelado and Retinto varieties (Table 4). In addition, genes that encode transcription factors involved in embryogenesis were also in previous studies of TRDLs in animals, mainly related to the mesoderm and neural system [23,63,64]. In this way, the Entrepelado variety showed an enriched GO linked to histone methylation generally associated with transcriptional repression and playing a vital role in the developing embryo [65,66]. Indeed, changes near histone genes might lead to the alteration of epigenetic marks and compromise embryo survival [67,68]. In this variety (Entrepelado), we could also find more GOs related to embryo development at the level of eye and heart development in the same address than some genes with at least one TRDL (described above). Given these results, it could be interesting to use the TRD phenomenon in assessing traits linked to reproduction and embryos in the future, as we commented above.

On the other hand, other GO biological processes were enriched separately in both Iberian varieties. In the Entrepelado variety, a GO related to the metabolism of calcium ion was significant, in line with a gene with a TRDL detected in this study (described above). The Iberian pig is known to show a fatty phenotype and lipid metabolism different from other commercial breeds, partly due to due to its *thrifty genotype*, and adaptive mechanisms to uneven feed availability [69]. In the Retinto variety, several biological processes associated with lipid metabolism and synthesis were found enriched, some of them related to cell membrane lipids, especially in the brain, such as sphingolipids and glycolipids [70]. These second lipids play fundamental roles in a variety of cellular processes and energy homeostasis [71]. In this way, some biological processes linked to nutrients and the sensory perception of chemical signaling, which are related to the TRDL found in an olfactory receptor gene (described above), were enriched in the Retinto variety. Unlike other studies, no common genes were found with previous gene regions affected by the TRDLs mapped (Table S4; [17,23]).

## 4. Conclusions

In conclusion, we have successfully modified a model to detect TRDLs with ungenotyped ancestors of different populations using the likelihood ratio test. Thanks to this model, we have screened the whole pig genome, and maternal TRDLs have been identified in both Entrepelado and Retinto Iberian varieties showing different and common points between them. No strong evidence of direct biological TRD effects was found, but there were possible biological processes affected in regions with TRDLs. The study of TRDLs could provide priceless insight into the genetic and evolutionary development of pig breeds, particularly in traditional breeds. These results may promote future research and the development of new strategies to further knowledge about TRD phenomena in the livestock industry.

## Figures and Tables

**Table 1:** Common maternal transmission ratio distortion loci (TRDLs) detected in both Iberian varieties.

ID Marker	Chr	Pos (Mb)	TRD_E	*q-*val_E	CallR_E	TRD_R	*q-*val_R	CallR_R	Gene with TRD Locus
rs339672482	2	12.698	−0.495	8.5 × 10^−36^	100.0	−0.495	1.8 × 10^−33^	100.0	ENSSSCG00000031496
rs343381067	2	14.467	−0.495	5.3 × 10^−34^	98.9	−0.409	1.4 × 10^−15^	99.4	ENSSSCG00000031436
rs335768329	4	83.114	−0.378	1.3 × 10^−12^	96.6	−0.495	8.8 × 10^−28^	94.5	ADCY10
rs81476539	6	72.737	−0.402	1.3 × 10^−16^	100.0	−0.322	4.1 × 10^−08^	100.0	
rs324128272	9	2.722	−0.473	4.8 × 10^−24^	99.4	−0.447	5.5 × 10^−23^	99.4	
rs346413844	9	11.795	−0.367	1.8 × 10^−10^	93.7	−0.346	5.2 × 10^−11^	97.8	
rs319847656	9	23.041	−0.453	4.3 × 10^−21^	99.4	−0.495	1.8 × 10^−33^	100.0	ENSSSCG00000049020
rs344225789	18	49.209	−0.439	5.5 × 10^−20^	99.4	−0.252	0.0131	99.4	
rs331690240	20	14.2345	−0.278	1.4 × 10^−05^	100.0	−0.26	1.2 × 10^−04^	99.4	ENSSSCG00000050465
rs343278080	20	26.079	−0.265	0.0231	100.0	−0.485	1.8 × 10^−29^	100.0	

Results from two Iberian pig varieties: Entrepelado (_E) and Retinto (_R). CallR: call rate. Pos: position. Q-val: *q*-value.

**Table 2:** Genes with no common maternal transmission ratio distortion loci (TRDLs) detected in both Entrepelado and Retinto varieties.

ID Marker	Chromosome	Position (b)	Gene with TRDL	Iberian Variety	Marker Call Rate
rs337916686	1	162,977,295	ATP8B1	Entrepelado	100.0
rs80864027	1	163,382,446	IGDCC4	Entrepelado	100.0
rs81305791	1	271,207,043	NUP214	Entrepelado	100.0
rs339672482	2	12,697,523	ENSSSCG00000031496	Entrepelado	100.0
rs343381067	2	14,467,327	ENSSSCG00000031436	Entrepelado	98.9
rs81221692	3	102,885,453	PRKD3	Entrepelado	100.0
rs335768329	4	83,113,768	ADCY10	Entrepelado	96.6
rs81385903	5	83,334,157	ANO4	Entrepelado	100.0
rs55618893	5	91,751,676	LUM	Entrepelado	100.0
rs320534160	7	1,148,169	GMDS	Entrepelado	100.0
rs80939667	7	7,368,476	GCNT2	Entrepelado	100.0
rs338044350	7	7,378,724	GCNT2	Entrepelado	100.0
rs327255612	8	4,896,260	EVC2	Entrepelado	100.0
rs81420408	9	2,210,850	SYT9	Entrepelado	100.0
rs319847656	9	23,040,656	ENSSSCG00000049020	Entrepelado	99.4
rs81262274	9	135,247,805	ENSSSCG00000031141	Entrepelado	100.0
rs335996850	10	24,233,693	RNPEP	Entrepelado	100.0
rs320095885	14	42,820,750	SGSM1	Entrepelado	100.0
rs80994847	14	43,934,858	SEZ6L	Entrepelado	100.0
rs334182161	18	3,613,926	DPP6	Entrepelado	100.0
rs327443567	18	17,971,591	ENSSSCG00000051610	Entrepelado	100.0
rs326744865	18	18,486,229	CPA1	Entrepelado	100.0
rs331690240	20	14,234,646	ENSSSCG00000050465	Entrepelado	100.0
rs320767193	20	120,168,429	ENSSSCG00000042120	Entrepelado	100.0
rs80947114	1	252,918,477	SUSD1	Retinto	100.0
rs333078973	1	268,840,777	CERCAM	Retinto	100.0
rs339672482	2	12,697,523	ENSSSCG00000031496	Retinto	100.0
rs343381067	2	14,467,327	ENSSSCG00000031436	Retinto	99.4
rs323787335	4	7,369,788	ZFAT	Retinto	100.0
rs335768329	4	83,113,768	ADCY10	Retinto	94.5
rs81414835	9	9,862,005	MAP6	Retinto	99.4
rs81414870	9	9,898,353	MAP6	Retinto	99.4
rs342178816	9	11,759,440	ENSSSCG00000014877	Retinto	100.0
rs319847656	9	23,040,656	ENSSSCG00000049020	Retinto	100.0
rs81280147	9	99,713,062	CD36	Retinto	100.0
rs331690240	20	14,234,646	ENSSSCG00000050465	Retinto	99.4

**Table 3:** The analysis of Gene Ontology (GO) biological process enrichment of the regions with common maternal transmission ratio distortion loci (TRDLs) detected in both Iberian varieties.

GO ID	Term	Genes Found	Fold Enrichment	*p*-Value Corrected
GO:0030307	Cell growth	1	56.78	0.0217
GO:0097061	Dendrite	1	56.78	0.0217
GO:0043542	Endothelial cell migration	1	45.42	0.026
GO:0048332	Mesoderm morphogenesis	1	22.71	0.0472
GO:0045216	Cell-cell junction	2	18.17	0.00622
GO:0050770	Axonogenesis	2	10.32	0.0174
GO:0010975	Neuron development	2	6.88	0.0359
GO:0000904	Cell morphogenesis	4	5.05	0.00881
GO:0000902	Cell morphogenesis	5	4.38	0.00617
GO:0009653	Morphogenesis	6	2.98	0.0163

**Table 4:** The analysis of Gene Ontology (GO) biological process enrichment of the regions with maternal transmission ratio distortion loci (TRDLs) detected separately in both Entrepelado and Retinto varieties.

GO ID	Term	Entrepelado Variety			Retinto Variety		
		Genes Found	Fold Enrichment	*p*-Value Corrected	Genes Found	Fold Enrichment	*p*-Value Corrected
GO:0060048	Cardiac muscle	3	8.91	0.00649			
GO:0048813	Dendrite	2	8.41	0.0294			
GO:0002088	Eye	3	7.57	0.00966			
GO:0050953	Light stimulus	3	7.57	0.00966			
GO:0034968	Histone-lysine methylation	3	7.21	0.0109			
GO:0050803	Synapse structure	3	6.88	0.0122	2	9.47	0.0216
GO:0045216	Cell-cell junction	3	6.06	0.0166	2	8.33	0.027
GO:0017156	Calcium	3	5.41	0.0219			
GO:0051592	Calcium	3	4.73	0.0301			
GO:0050770	Axonogenesis	4	4.59	0.014	3	7.1	0.0101
GO:0015850	Compound transport	4	4.49	0.015			
GO:0055002	Striated muscle	3	4.45	0.0348			
GO:0010975	Neuron development	5	3.82	0.0123	3	4.74	0.0281
GO:0099504	Synapse structure	4	3.11	0.0453			
GO:0000904	Cell morphogenesis	8	2.24	0.031	5	2.89	0.0322
GO:0009653	Morphogenesis	19	2.1	0.00348	7	1.6	0.218
GO:0000902	Cell morphogenesis	10	1.95	0.0432	6	2.41	0.0416
GO:0030307	Cell growth				1	26.05	0.0466
GO:0097061	Dendrite				1	26.05	0.0466
GO:0009247	Lipid biosynthesis				4	13.02	0.000369
GO:0044255	Lipid metabolism				9	3.14	0.00285
GO:0007606	Chemical stimulus				8	3.35	0.0033
GO:0046467	Lipid biosynthesis				4	6.72	0.00361
GO:0030148	Lipid biosynthesis				3	8.93	0.0056
GO:0007186	Receptor				16	1.94	0.0117
GO:0031623	Receptor				2	10.42	0.0183
GO:0007584	Response to nutrient				1	26.05	0.0466
GO:0015909	Lipid transport				1	26.05	0.0466

## Supplementary Materials

The following are available online at https://www.mdpi.com/2073-4425/11/9/1050/s1, Table S1: Single-nucleotide polymorphisms analyzed in both Entrepelado (E) and Retinto (F) families, Table S2: Transmission ratio distortion (TRD) loci detected in Entrepelado Iberian families, Table S3: Transmission ratio distortion (TRD) loci detected in Retinto Iberian families and Table S4: Genes belonging to significant Gene Ontology (GO) biological processes identified in the regions with maternal transmission ratio distortion loci (TRDLs) separately in both Entrepelado and Retinto varieties.
